# Genetic and Epigenetic Traits as Biomarkers in Colorectal Cancer

**DOI:** 10.3390/ijms12129426

**Published:** 2011-12-16

**Authors:** Marianne Berg, Kjetil Søreide

**Affiliations:** 1 Department of Surgery, Stavanger University Hospital, P O Box 8100, N-4068 Stavanger, Norway; 2 Department of Surgical Sciences, University of Bergen, 5021 Bergen, Norway; E-Mail: ksoreide@mac.com

**Keywords:** biomarker, colorectal cancer, mutations, epigenetics, microsatellite instability, chromosomal instability, risk marker, diagnostic marker, prognostic marker, predictive marker

## Abstract

Colorectal cancer is a major health burden, and a leading cause of cancer-related deaths in industrialized countries. The steady improvements in surgery and chemotherapy have improved survival, but the ability to identify high- and low-risk patients is still somewhat poor. Molecular biology has, over the years, given insight into basic principles of colorectal cancer initiation and development. These findings include aberrations increasing risk of tumor development, genetic changes associated with the stepwise progression of the disease, and errors predicting response to a specific treatment. Potential biomarkers in colorectal cancer are extensively studied, and how the molecular aberrations relate to clinical features. Yet, little of this knowledge has been possible to transfer into clinical practice. In this review, an overview of colorectal cancer genetics will be given, as well as how aberrations found in this tumor type are proposed as biomarkers for risk prediction, as diagnostic tools, for prognosis or prediction of treatment outcome.

## 1. Introduction

Colorectal cancer (CRC) is a major health burden in the western world. The disease normally develops from a benign polyp through an adenoma with dysplasia into a carcinoma with metastatic potential. The high incidence combined with high mortality from the disease if diagnosed at a late stage, signifies the need for better diagnostic, prognostic and predictive tools. The emergence of knowledge on the molecular level has gained insight in causes for initiation and progression of tumor development. This knowledge has also revealed the complexity and heterogeneity of the disease, explaining why few biomarkers are in routine clinical use.

Cancers arise as a result of genetic and epigenetic alterations accumulating in a cell [1]. These changes lead to dysregulation of fine-tuned pathways, and thereby disturb the normal proliferation and growth of the cell. Predominantly, colorectal cancer arises sporadically, although a smaller subgroup arises either as a result of inherited mutations, or as a result of inflammatory bowel disease (IBD; Crohn’s disease or ulcerative colitis).

Roughly, three molecular subtypes of colorectal carcinomas (CRC) are described based on molecular characteristics of the tumor; microsatellite instability (MSI), chromosomal instability (CIN), and CpG island methylator phenotype (CIMP). In addition, all of these three subgroups have mutations in protein coding genes, as well as impaired gene functions, and changes in expression of microRNAs (miRNA) that results in changes in gene expression.

Patient response to treatment is diverse, even if their disease seems similar when evaluating clinicopathological parameters. There is increasing evidence that treatment response is dependent on the normal genetic background of the individual in addition to aberrations in the tumor itself. Mapping the genetic defects in a patient tumor is yet of limited clinical importance, as the prognostic and predictive value is scarce. However, a large body of information about genetic aberrations underlying CRC has revealed complex and heterogeneous mechanisms underlying the occurrence of disease. This information is of the essence for understanding the disease behavior and related clinical outcome, and especially for future improvements in treatment and survival for the patients.

The current review article addresses some of the key concepts in CRC development, and outlines how genetic and epigenetic aberrations can be utilized as biomarkers for the disease in the future.

## 2. Phenotypic Subgroups of CRC

Chromosomal aberrations and aneuploidy are known as a hallmark of solid tumors. In colorectal cancers the large portion of the tumors display numerical chromosomal alterations, referred to as *chromosomal instability* (CIN) [2]. These CIN tumors have chromosomal composition which changes at a higher rate compared to normal cells, and recurrent gains and losses seem to affect chromosomes in a non-random manner [3]. The underlying cause(s) of CIN is to date not known, but alterations in mechanisms associated with chromosome segregation during mitosis are suggested [4,5]. The tumors in which mutations in microsatellites are demonstrated are referred to as *microsatellite instabile* (MSI) [6]. Tumors of the MSI phenotype have defects in the mismatch machinery, leaving errors introduced during replication unrepaired. Microsatellites are repetitive units, and therefore more prone to errors, and exist in both protein-coding and non-coding regions of the DNA. Deficient effect of mismatch repair genes has been found to cause this effect, either due to mutations in or as a result of hypermethylation of the promoter of these genes. Tumors harboring the latter of these aberrations have changes in the normal promoter methylation pattern. Methylation is chemical modification of DNA that leads to gene expression changes. This phenotype was discovered somewhat later than the CIN and MSI phenotypes, and named CpG island methylator phenotype (CIMP) [7,8]. As for the CIN phenotype, the underlying cause for CIMP is not revealed.

Initially, the CIN and MSI phenotype was thought of as mutually exclusive, but later found to be partly overlapping. The CIMP phenotype is largely overlapping with the MSI phenotype, and to some extent with CIN tumors. There is a small subgroup of tumors in which none of the phenotypes are detected, referred to as triple negative [9].

## 3. Biomolecules

The constant development and refinement of molecular techniques and knowledge about traits have increased our understanding of the human genome and its complexity in general, as well as cancer-specific aberrations. The total DNA content in a cell is referred to as the genome. The genes encoded by the genome are composed of both introns and exons, exons being the mRNA-encoding entities. The total content of mRNA in a cell at a given time point is referred to as the transcriptome. MicroRNAs (miRNA) are short RNA molecules that bind to complementary mRNA molecules, hindering the translation of the mRNA into a protein. In recent years both complete cancer genomes, transcriptomes, and exomes have been sequenced [10–12], DNA methylation profiles have been used to subgroup colorectal carcinomas [13], and even nucleic molecules such as microRNAs (miRNA) have been shown to play a role in cancer [14,15]. As non-protein-coding transcripts have been conserved throughout evolution, indicates that crucial functions exist for these molecules. For example, microRNAs (miRNAs) have been found to modulate several cellular processes [16]. The protein classes of RNA-binding proteins include essential regulators of miRNA biogenesis, turnover and activity. RNA-RNA and protein-RNA interactions are essential for post-transcriptional regulation in normal development and may be deregulated in disease. DNA, mRNA and miRNA are released and circulate in the blood [17]. Changes in the levels and types of circulating nucleic acids have been associated with tumor burden and malignant progression. Consequently, their potential role as markers of disease or risk for cancer is currently intensively investigated.

Faster and cheaper sequencing technology has made comparison of tumor exomes from patients with the same tumor type possible, helping the identification of cancer-driving mutations [12]. Also, comparison of primary tumor and the associated distant metastasis has aided the search for genes, which is important for the metastatic process or the progression of disease [18].

## 4. Adenoma Carcinoma Sequence

In colorectal cancer, adenomas are considered the most important precursor lesion for carcinomas, although a subgroup of hyperplastic polyps have also been shown to have malignant potential [19]. By the age of 70 years around 50% of the population has one or several adenomas presented in the colon. However, not all adenomas progress to carcinomas. The adenoma-carcinoma sequence suggests that specific mutations occur at specific stages, in order to transform the cell to a carcinoma with a metastatic potential. These mutations affect genes and pathways important for regulation of cell growth and differentiation.

**Figure 1:**
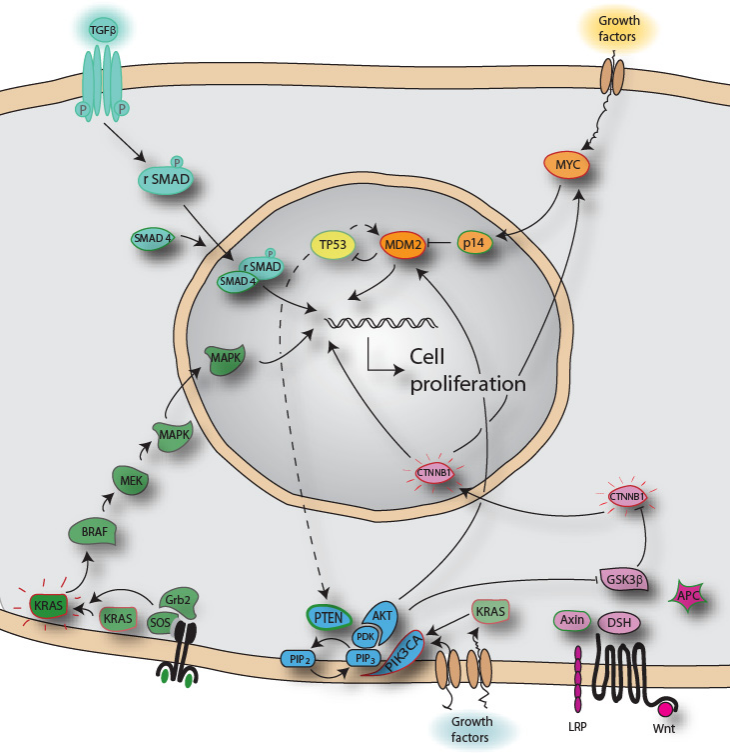
Signaling pathways frequently found to be changed in colorectal cancer [25].

The WNT pathway increases the proliferation rate in a cell when active, and is known as an early event in the adenoma-carcinoma-sequence [20]. The *APC* gene is thought to be the initial event transforming a normal cell into an adenoma, and is found in ~80% of all colorectal carcinomas. If *APC* is mutated, the WNT signaling pathway is constantly on, even if WNT signal is absent, and the cell proliferates, Figure 1. Mutation in the oncogene *KRAS* is another aberration seen both frequently (~40%), and early in the transformation of normal cells. Mutations in the oncogene *BRAF*, yet another gene in the MAPK pathway, are seen early in malignant transformation. Both *KRAS* and *BRAF* mutations will increase the proliferation rate in the cell, comparable to mutations in *APC*. The TGFb pathway has been shown to be abrogated later in the development, transforming an intermediate adenoma to a late adenoma. Mutations in *SMAD4*, *TGFBR2* or deletions at 18q are observed [21]. Mutations in the tumor suppressor gene *TP53*, or loss of 17p where *TP53* is located, are observed in more than 50% of colorectal tumors, and as late events in CRC development. This high frequency of *TP53* inactivity found in carcinomas, but not in adenomas, suggests *TP53* to be pivotal for the malignant transformation of the cell.

A subgroup of hyperplastic polyps, sessile serrated adenomas, were previously regarded as *not* giving increased risk of colorectal carcinoma development [19]. An equivalent to the adenoma-carcinoma sequence has been suggested for these adenomas [22], of which an activating mutation in the *BRAF* gene is regarded as the initiating event of the malignant transformation. Furthermore, methylation of promoter regions resulting in epigenetic silencing of a number of genes is observed in these lesions, the so-called CIMP phenotype. The *MLH1* gene is one of the genes frequently shown to be methylated, and abrogation of the normal function of *MLH1* will eventually cause MSI tumors. The observation that sessile serrated adenomas gives rise to CIMP and MSI tumors are supported by the fact that these polyps are most frequently found in the right colon [23,24].

Genes found to be mutated in cancer cells have been defined as either proto-oncogenes or tumor suppressor genes. The proto-oncogenes are promoting cell growth, and when mutated the proliferation rate in the cell is high even in the absence of growth signals. In contrast, mutant tumor suppressor genes are deprived of their cell cycle regulatory function, resulting in cell proliferation even if severe damages are introduced in the cell–thus, the normal regulation and balance between apoptosis and proliferation is skewed towards an uncontrolled proliferative state with loss of cell death. However, several more hallmarks are needed for a cancer cell to sustain its growth, expand, invade and ultimately seed and soil its metastatic cells in distant organ sites.

Sjoblom *et al.* [26] and Wood *et al*. [27] reported that around 70 genes on average are mutated in a colorectal tumor sample, of which ~10 are likely to drive tumorigenesis, based on sequencing of 13,023 protein coding genes. Later, Timmermann *et al.* [12] performed exome sequencing (16,755 RefSeq genes) of MSI and microsatellite stable (MSS) CRCs, and compared the mutation pattern in each tumor to normal colonic mucosa from the same patient. This highlighted specific mutation patterns in the two CRC subtypes, and 359 and 45 functionally significant mutations were reported in MSI and MSS tumors, respectively, which confirms MSI as the mutator phenotype. Furthermore, the *BMPR1A* gene, mutated in germline cells in juvenile polyposis, was found to be mutated in both MSI and MSS tumors, indicating that this gene might also have an important function in sporadic CRC development.

## 5. Biomarkers

The growing insight in molecular mechanisms of cancers has increased the expectations of these aberrations and compounds to be used as biomarkers. A plethora of molecules have been suggested as markers for risk, diagnosis, prognosis and treatment response, Table 1. But so far, mutations in the oncogene *KRAS* is the only biomarker in routine clinical use in CRC, validated to have information predicting response to treatment, [28].

### 5.1. Markers Aiding in Prediction of Risk

Familial syndromes are estimated to cause 25% of colorectal cancer, but only around 5% of colorectal cancers are identified to have a known genetic defect [57]. The best described syndromes are Lynch syndrome (aka Hereditary Non-Polyposis Colorectal Cancer, HNPCC), developed on a background of an inherited germline defect DNA-mismatch repair systems leading to widespread MSI, and the Familial Adenomatous Polyposis (FAP) syndrome, caused by germline mutations in the APC gene. HNPCC is clinically associated with other cancers such as gastric and endometric cancer but no widespread development of colonic polyps despite cancer development at an early age (<50 years). FAP patients however, develop polyps in hundreds and thousands, necessitating total colectomy often at or before the age of 20 to avoid malignant transformation. Although most patients do not fall into one of these two hereditary categories, many patients may have family members with the disease, despite no clear cut genetic association found. However, to date, several genes are known to harbor mutations causing hereditary syndromes involving colorectal cancer [58–60].

In addition to inherited mutations, a number of chromosomal locations and single nucleotide polymorphisms (SNPs) have been suggested as having increased risk for CRC development, but few have been validated in larger cohorts. However, in 8q24 [34–37], 15q13.3 [29,37,38], *SMAD7* [32,37,39,40], and LOC120376 [33,37,39], SNPs have been validated, and each of them shown to be associated with increased risk of CRC development. Furthermore, if the SNP is located in a miRNA target site it might interfere binding, resulting in changes in gene and protein expression. More aberrations, especially those causing disease in a recessive manner, are predicted to be discovered in the future.

### 5.2. Diagnostic Markers

Early detection of colorectal cancer is pivotal for a good outcome of the disease. The fecal occult blood test (FOBT) is a non-invasive diagnostic test based on detection of blood in the feces. The test has shown low sensitivity and specificity, especially for early disease stages, as not all cancers bleed, and bleeding can be caused by other conditions than cancer. Whether the test reduces mortality is debated [61], although at least three large randomized controlled trials have demonstrated a reduction in cancer-specific mortality with the use of FOBT. A further drawback is the need for invasive follow up (usually by colonoscopy) for positive findings. Screening populations using colonoscopy or sigmoidoscopy is expensive and unpleasant for the individual, which reduces the compliance among patients. Further colonoscopy is associated with a small but relevant risk for adverse event (perforation during the procedure) which may indeed be life-threatening. “Virtual colonography” or CT colonography has been proposed as a non-invasive alternative, but introduces the risk of radiation exposure, and again, a number of follow up test will be needed to investigate positive findings. Consequently, the need for better and preferably less invasive tests are warranted. Testing for changes in methylation pattern in feces and blood samples have shown improvements in sensitivity and specificity compared to the FOBT test [61,62]. The commercially available test ColoSure^TM^ examines methylation in the *Vimentin* gene in feces samples, for early diagnosis of CRC [63,64]. Although its sensitivity (77%) and specificity (83%) is enhanced compared to FOBT, detection rates have not been assessed in a normal population and its clinical utility is therefore not established [42]. Panels of methylated genes have been recognized in feces samples, in which increased sensitivity and specificity of colorectal tumors are identified [65,66].

The presence of methylation of the *Septin9* (*SEPT9*) gene has been shown to be highly correlated to occurrence of colorectal tumor cells. [67] Testing for methylation of *SEPT9* in plasma has been commercialized as ColoVantage^TM^.

Using dysregulation of the expression of circulating miRNAs as markers for early diagnosis has gained more attention over the last years. In a study by Ng *et al*., elevated levels of miR-17-3p and miR-92 were reported to be statistically significant compared to the levels in healthy individuals, and also compared to gastric cancer and inflammatory bowel disease [44].

### 5.3. Prognostic Markers

Even if meta-analyses have clearly shown a better prognosis of MSI cancers compared to CIN [68], clinical and histopathological data is still used as the main prognosticator and basis for treatment regime in CRC. The outcome of patients at disease stage II and III is however difficult to predict. Especially, for stage II cancers, defined on the basis of *not* finding metastases in lymph nodes, which could be erroneous, as a pathologist will not have the possibility to investigate all cells in all resected lymph nodes. Gene expression of *GCC* in tissue from lymph nodes has shown a good correlation to disease outcome, refining correct staging of stage II patients. The test is available as a commercial test manufactured as Previstage [51]. A more precise separation of stage II and III CRCs in high- and low risk groups have been obtained using mRNA expression profiles of 18 genes, commercialized as ColoPrint, Table 2 [46]. Yet another commercially available test, (OncoType DX), uses mRNA expression profile of 12 genes in order to indicate a risk of relapse in stage II patients [48]. Also, expression of miR-21 has been shown to be correlated to poor survival and therapeutic outcome in stage II and III CRCs [52], as well as being an independent predictor of overall survival in CRC [53].

### 5.4. Predictive Markers

The *KRAS* proto-oncogene is a molecular switch that controls cellular proliferation and differentiation, and its activation through EGFR is of essence for its proliferative effect in CRC. Overexpression, amplification and mutations of EGFR, resulting in signalling through the MAPK and PI3K pathways, are frequent in CRC, and it is therefore a good target for treatment of CRC. For metastatic colorectal cancer (mCRC) treatment by EGFR-targeted drugs is one of few therapeutic alternatives. However, when EGFRs downstream effector KRAS is mutated, it becomes constitutively active, the MAPK pathway is constantly switched on, and thus, treatment targeting EGFR will fail. Furthermore, only ~30% of the wild-type *KRAS* patients benefit from this treatment [69]. This indicates that other downstream effectors of the MAPK pathway or the PI3K pathway might have activating mutations. There are increasing evidences that the V600E mutation in the *BRAF* gene also inhibits effective treatment with anti-EGFR-therapies [69,70]. Furthermore, mutations in the *PIK3CA* gene, and overexpression of PTEN have also been associated with lack of response, and a combined analysis of these four genes would predict outcome in 70% of the patients [71]. Another theory that has been suggested for the lack of effect in *KRAS* wt samples, is heterogeneity within the tumor and between primary tumor and metastasis [72,73].

A panel of markers has recently been published to be associated with resistance to neoadjuvant chemoradiation therapy in rectal cancer [55]. The mutation profile of 23 genes was studied, and correlated to patient response to chemoradiation determined, in order to identify which patients will benefit from this treatment. Using a subset of four markers (*TP53*, *KRAS*, *CCND1* and *MTHFR*), they report a specificity of 97%, and a sensitivity of 52% for predicting patients without pathologic complete response. This implies a possibility to predict which patients will be resistant to chemoradiation therapy, and spare these patients unwanted side-effects [41].

Despite the fact that the knowledge of miRNA’s existence is few than 20 years, their function has been linked to anticancer chemotherapy in model systems. Resistance to 5-FU and methotrexate has been associated with expression of the miRNA miR-140 in the HCT116 cell line [56].

## 6. Conclusions and the Way Forward

Colorectal cancer most frequently develops sporadically, and at an increasing rate also in young patients (<50 years), although the highest prevalence still occurs in those aged 60–70 years. As patients having a localized disease stage at time of surgery have a very favorable prognosis, the early detection of the disease represents a compelling opportunity to reduce the disease burden. Clinical symptoms are non-specific, or unnoticeable at early disease stages, therefore there is a need for modes of early detection to reduce morbidity and mortality of the disease. Colonoscopy is still the golden standard in detecting aberrant lesions, but expensive and unpleasant for the patient. The excitement regarding blood and stool-based tests has therefore been great, but to date these tests have shown poor sensitivity and specificity compared to colonoscopy.

Over the last decade, large-scale technology has been implemented in the search for deviations underlying diseases. Despite initial optimism regarding the output from these analyses, findings have not revolutionized the field of colorectal cancer yet. First and foremost this technology is hypothesis-generating. Findings must be validated, e.g., with different downstream analysis tools; in new patient sample sets; and, with other methods, before robust implementation of biomarkers in the clinic can be recommended. The most recent advance in the large-scale technology field is the next-generation sequencing methodology. Identification of variants and mutations in an individual tumor will add information to the type of cancer and refine classification of the specific tumor. Also, a comparison of the whole genome sequence of normal cells from the tumor bearing individual will reveal tumor-specific aberrations. In total this can be utilized as prognostic and predictive tools in a clinical setting, and as information supporting a more personalized treatment. However, as knowledge about additional mechanisms of impairing cell signaling, such as molecular modification of DNA (e.g., methylation), regulation of mRNA and protein generation (e.g., miRNA), redundant pathways and genetic mechanisms has emerged, the realization that mutation screening solely will probably not reveal outcome of disease and treatment has also dawned.

Several genes and pathways affected by changes have been identified in colorectal tumors, and the knowledge about initiation and progression of the disease is extensive. Also, the number of published articles aiming at using this knowledge as biomarkers is numerous. However, the implementation of this knowledge into clinical practice is so far limited. To date, testing the gene *KRAS* for activating mutations is in routine clinical use, as it has been shown to have predictive information in relation to treatment with antibodies against the EGFR-receptor. Findings reported in the literature support additional testing of *BRAF*, *PTEN* and *PIK3CA* before anti-EGFR treatment in metastatic colorectal cancer. On the other hand, there is an increasing panel of treatments that have been designed to target specific genes and pathways, which further highlights the role of normally functioning genes in order for the drug to have the desired effect in the patient.

As more biomarkers are identified and validated it is anticipated that these will be used more extensively in clinical decision making. Optimization of tools to predict the risk for developing cancer, diagnose a disease at an early stage, give a prognosis that is as correct as possible, and predict treatment response in the patient, is of invaluable significance. First and foremost this applies to the patient itself, but also to the health personnel, and in a socioeconomic perspective.

## Supplementary Information



## Figures and Tables

**Table 1:** Type of biomarkers, and examples of biomarkers in use/suggested for use in colorectal cancer.

Type of Biomarker	Objective for use	Biological marker	References
Risk stratification	Assess the likelihood that cancers will develop	*APC*, *AXIN2*, *BMPR1A*, *SMAD4*, *MUTYH*, *MSH2*, *MLH1*, *MSH6*, *PMS2*, *STK11*, *PTEN*, *EPCAM*, 8q24, 15q13.3, SMAD7, LOC120376	[29–40]
Screening	Detect cancers in the asymptomatic population	Stool tests, blood based tests	[41]
Diagnosis	Definitively establish the presence of cancer	Vimentin (ColoSure *), SEPT9 (ColoVantage *), miR-17-3p, miR-92	[42–44]
Classification	Classify patients by disease subset	MSI, CIN, CIMP	
Prognosis	Predict the probable outcome of cancer regardless of therapy	MSI, 18-gene signature (ColoPrint *), 12-gene signature (OncoType DX *), GCC expression (Previstage *), miR-21	[45–53]
Prediction/treatment stratification	Predict response to particular therapies and choose the drug that is mostly likely to yield a favorable response in a given patient	EGFR, *KRAS*, *BRAF*, *PIK3CA*, *PTEN*, *TP53*, miR-140 Panel (*TP53*, *KRAS*, *CCDN1*, *MTHFR*)	[54–56]

Abbreviations; MSI: microsatellite instability, CIN: Chromosomal instability, CIMP: CpG island methylator phenotype;

* Details of biomarkers used are specified in Supplementary Table 1.

**Table 2:** Details of commercially available tests.

Test name	Biological material	Biomarker(s)
ColoSure^TM^	Methylated DNA in feces	*Vimentin*
ColoVantage®	Methylated DNA in plasma	*SEPT9*
ColoPrint®	mRNA expression in tumor tissue	*MCTP1*, *LAMA3*, *CTSC*, *PYROX D1*, *EDEM1*, *IL2RB*, *ZNF697*, *SLC6A11*, *IL2RA*, *CYFIP2*, *PIM3*, *LIF*, *PLIN3*, *HSD3B1*, *ZBED4*, *PPARA*, *THNSL2*, *CA4388O2*
OncoType DX®	mRNA expression in tumor tissue	*Ki-67*, *C-MYC*, *MYBL2*, *FAP*, *BGN*, *INHBA*, *GADD45B*, *ATP5E*, *PGK1*, *GPX1*, *UBB*, *VDAC2*
Previstage^TM^	mRNA expression in lymph node tissue	*GCC (GUCY2C)*
