# Supplementary material for: Genetic and Epigenetic Traits as Biomarkers in Colorectal Cancer
Source: Int J Mol Sci. 2011 Dec 16;12(12):9426–39. doi: 10.3390/ijms12129426 (PMC3257138; doi:10.3390/ijms12129426)
Supplement: Supplementary file 1 [file ijms-12-09426-s001.pdf]

Supplementary Information

Table 1S. Details of commercially available tests.

Test name	Biological material	Biomarker(s)
ColoSure™	Methylated DNA in feces	*Vimentin*
ColoVantage®	Methylated DNA in plasma	*SEPT9*
ColoPrint®	mRNA expression in tumor tissue	*MCTP1, LAMA3, CTSC, PYROX D1, EDEM1, IL2RB, ZNF697, SLC6A11, IL2RA, CYFIP2, PIM3, LIF, PLIN3, HSD3B1, ZBED4, PPARA, THNSL2, CA4388O2*
OncoType DX®	mRNA expression in tumor tissue	*Ki-67, C-MYC, MYBL2, FAP, BGN, INHBA, GADD45B, ATP5E, PGK1, GPX1, UBB, VDAC2*
Previstage™	mRNA expression in lymph node tissue	*GCC (GUCY2C)*

© 2011 by the authors; licensee MDPI, Basel, Switzerland. This article is an open access article distributed under the terms and conditions of the Creative Commons Attribution license (http://creativecommons.org/licenses/by/3.0/).
